# Mr. Proctor, on the Concentration of Vinegar

**Published:** 1799-09

**Authors:** John Proctor

**Affiliations:** Newcastle


### *To the Editors of the Medical and Physical Journal.*

Gentlemen,

The insertion of my last paper in your valuable publication was particularly gratifying, and encourages me to trouble you again with a few remarks on the concentration of vinegar, and on some pharmaceutical preparations formed from this acid, which, if they appear of sufficient importance, I have no doubt of your honouring them with your notice.

I am, Gentlemen,

Your obliged humble servant,

Newcastle, *August 6th*, 1799. JOHN PROCTOR, Jun.

The utility of this acetous acid, both as an article of diet and of the Materia Medica, renders an easy method of obtaining it pure, and tolerably concentrated, of considerable value. When it is not contaminated with the mineral acids, it may be deprived of a considerable portion of its aqueous part by freezing; but this is a process which cannot at all times be accomplished, nor even when it can, is it sufficient to deprive it of the other acids which may be mixed with it; and all the common kinds of vinegar sold in the shops are more or less adulterated with sulphuric acid. Distillation frees it perfectly from this acid; but distilled vinegar, being diluted with water, is too weak for many purposes; and where the deprivation of the colouring and extractive matters is not of much consequence, this operation may be dispensed with.

By diſſolving pure chalk in a given quantity of vinegar till it be ſaturated, we obtain a ſolution of acetated lime, from which we may ſeparate as much of the watery part as we pleaſe by evaporation; and afterwards by decompoſing this ſolution, by means of the ſulphuric acid, the acetous acid may be thus obtained of any degree of ſtrength required.

When pure vinegar cannot be obtained for this purpoſe, even ſuch as is adulterated with ſulphuric acid can be purified and concentrated by this proceſs with equal ſucceſs. The chalk neutralizes the mineral acid, and forms gypſum, which being inſoluble, is precipitated, and the acetated lime remains in ſolution. This is to be poured off, and evaporated to two thirds, or leſs; as much ſulphuric acid is then to be added as will ſeparate all the calcareous earth, adding it by little at a time, and taking care to avoid an exceſs of it, which may be prevented by trying a little of the vinegar from time to time, with a farther addition of ſulphuric acid, in a ſeparate veſſel; if it is not rendered turbid, it will be neceſſary to aſcertain, with another portion of it, whether the ſulphuric acid does not prevail, by adding to it a few drops of vinegar of litharge, or of a ſolution of ceruſſa acetata. When the point of ſaturation is finally adjuſted, and the gypſum ſeparated by depoſition, the ſupernatant liquor may be poured off; and it will be found to be a very pure and tolerably concentrated acetous acid.

It is however to be obſerved, that in this proceſs the flavour of the vinegar will in ſome meaſure be altered, if not loſt, by the evaporation of the volatile matter in which it reſides; but it might probably be reſtored, or ſome other flavour equally agreeable be communicated to it, by a ſhort digeſtion on wine-lees.

Diſtilled vinegar, as well as that which has not been diſtilled, may be treated in the ſame manner, if it be required to obtain the acid free from the ſavonulous extract combined with it in its crude ſtate. There is no doubt but vinegar thus concentrated might be advantageouſly employed as a powerful antiſeptic in pickling, and its piquancy as a ſauce be conſiderably increaſed by this proceſs.

The London College have given a method of preparing a concentrated acetous acid, by diſtilling verdigris per ſe, which does not appear, however, to be introduced for any other purpoſe than the making of the hydrarg. acet. Either from the apprehenſion of this acid containing copper, or on account of its high price, it has been ſince laid aſide, and another method of preparing the hydrarg. acet. ſubſtituted in the room of the former one, viz. decompoſing a ſolution of quickſilver in nitrous acid, by means of acetated kali. But the uſing of this neutral ſalt is ſtill a needleſs expence, as the precipitate obtained from the nitrous ſolution by the vegetable alkali can be diſſolved in diſtilled vinegar by boiling them together, when the ſame cryſtals of acetated quickſilver are depoſited on cooling, as are obtained by the other more complicated plicated and expenfive proceffes. Hence it is evident that the pure acid has a greater attraction for the metallic precipitate, than for caloric or water; fo that it is only the water of the folution that is evaporated, and which, if collected in a condenfed ftate, would be found totally free from any acid impregnation.

We may obferve in a number of chemical operations, how much the order of affinities is regulated by circumftances which require particular attention. Thus, water impregnated with carbonic acid gas foon becomes vapid by expofure to the ordinary temperature of the atmofphere, while its combination with fixed alkalies refifts the heat of boiling water; for the fubcarbonate of potafh (falt of tartar) is capable of decompofing concrete ammonia, and combining with the carbonic acid to the point of faturation, and the volatile alkali may then be diffipated by evaporation.

In the preparation of the aq. lithargyri acet. the College direct the ufe of diftilled vinegar, which is hardly neceffary: if the common kinds of vinegar be ufed, as ftrong a folution may be obtained, and every way equally fit for ufe, as that made with diftilled vinegar. During the boiling, the fulphuric acid is all feparated from it, forming with part of the litharge a fulphat of lead which is precipitated, and the pure acetous acid is left at liberty to faturate itfelf with the remainder. Inftead of boiling the folution down to a certain quantity, it would be better to bring it to an accurate ftandard by weight; as fuppofing a long narrow-necked bottle, which, when full, held 1. 0 of water, fhould hold 1. 3 of the aq. lith. acet.

When the aq. veg. min. is made with hard water, a partial decompofition of the vinegar of litharge takes place, which is not even prevented by ufing diftilled water, becaufe part of the lead is not fufficiently oxydated to keep it diffolved. If inftead, therefore, of adding the proof fpirit (which rather ferves to increafe the decompofition), as much diftilled vinegar were ufed in its place, a permanently tranfparent folution would be had, and of a conftantly uniform ftrength. Sugar of lead and ceruffe feem to bear the fame relation to each other, as corrofive fublimate and calomel. See CRELL's *Chemical Journal*, vol. iii. p. 8.

The foregoing remarks apply with equal propriety, and much more forcibly to the folution of antimonial tartar, both in water and in wine; part of the antimonial oxyd being depofited in the fame manner from both folvends. I would therefore recommend the rejection of the vin. tart. antim. and a fubftitution in its place of a given quantity of emetic tartar diffolved in water, with the addition of as much of a faturated folution of cryftals of tartar as may be found fufficient to prevent any precipitation. I am perfuaded that the objections to this active medicine, on account of the uncertainty of its ftrength, would, by this manner of preparing it, be confiderably done away, and that there would be lefs occafion for introducing other formulæ, neither fuperior in efficacy, nor fimpler in compofition.

